# Transdiagnostic Risk and Protective Factors for Psychopathology in Young People: Systematic Review Protocol

**DOI:** 10.2196/19779

**Published:** 2020-08-20

**Authors:** Samantha Jane Lynch, Matthew Sunderland, Nicola Claire Newton, Cath Chapman

**Affiliations:** 1 The Matilda Centre for Research in Mental Health and Substance Use University of Sydney Sydney Australia

**Keywords:** psychopathology, mental health, adolescent, young people, transdiagnostic, risk factors, protective factors, systematic review, protocol

## Abstract

**Background:**

Mental and substance use disorders are among the leading causes of burden of disease worldwide, with risk of onset peaking between the ages of 13 and 24 years. Comorbidity is also common among young people and complicates research, diagnosis and assessment, and clinical decision making. There is increasing support for empirically derived models of psychopathology that overcome issues of comorbidity and provide a transdiagnostic framework for investigating the specificity and generality of risk and protective factors for psychopathology.

**Objective:**

This systematic review aims to identify transdiagnostic risk and protective factors for psychopathology in young people by synthesizing and evaluating findings from research investigating empirically based models of psychopathology.

**Methods:**

Searches will be conducted in Medline, EMBASE, and PsycINFO databases. Reference lists of selected articles will also be hand searched for other relevant publications. All studies will be screened against eligibility criteria designed to identify studies that examined empirical models of psychopathology in relation to risk and/or protective factors in young people with a mean age between 10 and 24 years. Study quality will be assessed using the Joanna Briggs Institute Critical Appraisal Checklists for Cohort Studies and Analytical Cross-Sectional Studies. Findings will be summarized in a narrative synthesis, and a meta-analysis will be conducted if sufficient data are available.

**Results:**

This review is ongoing. At the time of submission, full-text screening was completed, and hand searching of selected articles was underway. Results are expected to be completed by the end of 2020.

**Conclusions:**

This protocol is for a systematic review of evidence for transdiagnostic risk and protective factors associated with empirically based models of psychopathology in young people. To our knowledge, the critical synthesis of this evidence will be the first to date and will provide a better understanding of the factors that contribute to the onset and maintenance of psychopathology in young people. Insights drawn from the review will provide critical new knowledge to improve the targeting of interventions to prevent or reduce mental health problems.

**Trial Registration:**

This systematic review is registered with PROSPERO (CRD42020161368) and is available via Open Science Framework.

**International Registered Report Identifier (IRRID):**

DERR1-10.2196/19779

## Introduction

Mental and substance use disorders are among the leading causes of burden of disease worldwide, and the mortality and morbidity of these disorders have not declined since 1990 [1]. These disorders often emerge during adolescence, with risk of onset heightened between the ages of 13 and 24 years [2,3]. A number of factors have been identified that increase (risk factors) or decrease (protective factors) the likelihood of young people experiencing mental health problems. Risk and protective factors help identify young people most at risk of developing mental disorders and guide intervention targets. Many risk and protective factors have been found to be associated with a number of different mental disorders [4]. However, it is unclear whether these associations are specific to certain mental disorders or transdiagnostic in nature.

Comorbidity among mental disorders is common, with estimates that up to two-thirds of adolescents with a mental disorder will also have at least one other mental disorder [3,5]. The prevalence of comorbidity makes diagnostic and treatment decision making complicated, as additional disorders can affect treatment outcome [6,7]. Furthermore, failing to account for comorbid mental disorders when investigating risk and protective factors could mean that relationships with mental disorders might be due to the compounding nature of overall psychopathology rather than any specific associations, hampering research, prevention, and treatment efforts.

Given the ubiquity of comorbidity, understanding risk and protective factors in relation to the development of mental disorders in young people is important for three reasons. First, comorbidity has been associated with greater symptom severity and poorer treatment outcomes [3,8]. Second, risk and protective factors may enhance identification and prediction of individuals with a greater likelihood of developing mental disorders [9]. Third, identification of the characteristics and processes that can be targeted and modified through intervention is critical to the development of efficacious prevention and treatment [10]. Much of the prior research investigating risk and protective factors has typically focused on associations with a single disorder or a single risk or protective factor [4]. As such, the relationships between the breadth of psychopathology and putative risk and protective factors are not clear, heralding the need for a different approach to examining these relationships.

### Empirical Models of Psychopathology

The categorical*,* prototypical approach to organizing mental disorders used in traditional classifications systems, such as ﻿the Diagnostic and Statistical Manual of Mental Disorders (DSM; now in its 5th edition) has a number of limitations, such as a lack of specificity as demonstrated by the prevalence of comorbidity [7]. In contrast, empirical models of psychopathology use a broad range of quantitative approaches to generate coherent arrangements of signs and symptoms of psychopathology and capture the high rates of psychiatric comorbidity [11]. What results is a quantitively organized framework that facilitates investigation of the specificity and generality of risk and protective factors for psychopathology that is not achievable with traditional classification systems [9,12,13]. Two empirical models have emerged in recent years and received increasing attention in the literature.

#### Hierarchical Dimensional Models

Hierarchical dimensional models, such as the Hierarchical Taxonomy of Psychopathology (HiTOP) model, propose latent factors that capture covariance among commonly comorbid disorders. Early examination of comorbidity among common childhood disorders suggested the presence of two latent factors: internalizing (eg, mood and anxiety disorders) and externalizing (eg, substance abuse and antisocial, oppositional, and impulsive related disorders) factors [3,11,14]. However, internalizing and externalizing have also consistently been found to be positively correlated, suggesting the presence of a higher-order latent factor [3,12,15].

According to the HiTOP model, this association represents a general factor of psychopathology (the “p” factor). The “p” factor sits at the apex of the hierarchical structure and is thought to capture a latent vulnerability to all mental disorders (see Kotov et al [6]). Efforts to expand the internalizing-externalizing model to cover the breadth of psychopathology have flourished over the last two decades. Additional spectra that sit below the “p” factor have also begun to emerge, such as thought disorder (or psychoticism), detachment (eg, histrionic, avoidant, dependent, and schizoid personality disorders), and somatoform dimensions. Beneath each of these spectra sit a number of lower order dimensions, and beneath these sit a number of even more specific components and traits. In this framework, transdiagnostic risk and protective factors may be uniquely associated with the “p” factor or specific spectra, such as internalizing or externalizing.

#### Network Models

Network theory proposes that disorders arise from dynamic relationships between symptoms, resulting in a network of connected symptoms [13]. Disorders can therefore be seen as systems of causally related symptoms, rather than manifestations of latent vulnerabilities. Factors outside of the psychopathology network form what is referred to as the external field and can influence or activate symptoms, which in turn promotes the activation of other symptoms in a cascading system leading to the onset and maintenance of mental disorders [16]. Transdiagnostic risk and protective factors are therefore components of the network that are external to symptoms but are connected to symptoms from many symptom groupings within the psychopathology network.

### Transdiagnostic Risk and Protective Factors

Two previous reviews have examined risk and protective factors in relation to internalizing and externalizing dimensions in children and adolescents; however, to our knowledge, no previous reviews have investigated other broadband dimensions [17,18]. A mega-analytic synthesis of child, family, school, community, and cultural risk and protective factors correlated with internalizing behaviors, externalizing behaviors, or both found 4 risk factors and 3 protective factors common to both internalizing and externalizing disorders [17]. Although this suggests that additional factors examined were specific to either internalizing or externalizing, it is unclear from the review whether the studies included examined both internalizing and externalizing disorders simultaneously, only one of these, or specific behaviors or disorders within those disorder groupings. Thus, it is not possible to draw any conclusions about whether any of the identified risk and protective factors are transdiagnostic or disorder-specific.

McMahon and colleagues [18] conducted a systematic review of studies examining the relationship between internalizing and externalizing symptoms and a range of stressors, such as exposure to violence, abuse, poverty, and parental divorce, with the aim of evaluating the specificity of stressors. However, the review found little evidence that individual stressors were associated with specific internalizing or externalizing outcomes, with the exception of an association between sexual abuse and internalizing or post-traumatic stress disorder symptoms. This suggests that most stressors examined were transdiagnostic across internalizing and externalizing disorders. However, while stressors may be transdiagnostic risk factors and useful for identifying young people at risk of developing mental health problems, further investigation is needed to identify factors that can be addressed and modified through intervention. Further, it is unknown whether these transdiagnostic associations hold across other domains of psychopathology, such as psychotic-related disorders.

Research that takes into account a broad range of disorders and comorbidity is necessary to identify transdiagnostic risk and protective factors. Identifying the risk and protective factors for psychopathology in young people that occur across traditional diagnostic categories is of great clinical significance. Such factors may be useful for more efficient prediction and early identification of psychopathology, as some may provide useful targets for reducing overall risk for psychopathology, thus preventing a variety of mental disorders from subsequently emerging [19].

### Review Aim

The aim of this systematic review is to identify transdiagnostic risk and protective factors for psychopathology in young people. This will be done by synthesizing and critically evaluating studies examining empirically based models of psychopathology.

## Methods

This protocol conforms to the Preferred Reporting Items for Systematic Reviews and Meta-Analysis Protocols (PRISMA-P) statement [20], which can be found in Multimedia Appendix 1, and is registered with PROSPERO (CRD42020161368). The protocol is also available via Open Science Framework [21].

### Eligibility Criteria

The Population Exposure Comparator Outcome (PECO) framework was used to develop the research question and eligibility criteria for this review [22].

#### Population

The population of interest will be young people between 10 and 24 years of age, as defined by the World Health Organization [23]. Studies where the mean age of participants falls between 10 and 24 years will be considered for inclusion.

#### Exposure

Studies that have examined variables such as genetic, neurobiological, cognitive, social, and environmental characteristics and their association with an empirically based model of psychopathology will be considered for inclusion.

#### Comparison

Studies with or without a comparison group will be considered for inclusion as the dimensional nature of psychopathology implicit within contemporary knowledge precludes the need for control groups.

#### Outcome

Psychopathology outcomes derived from empirically based models of at least two broad groups of signs or symptoms, such as internalizing, externalizing, or thought disorders, will be included. Quantitative approaches typically used to organize signs and symptoms of psychopathology include factor analytic, class-based, and network approaches. Studies where validated measures of internalizing and externalizing have been used will also be included where findings for both dimensions have been reported.

#### Studies

Longitudinal and cross-sectional studies examining risk and protective factors associated with psychopathology in young people will be eligible. Although longitudinal studies provide stronger evidence for causation, cross-sectional studies will be included because they may help identify characteristics needing further research.

Studies must be peer-reviewed, be in English, and report original empirical findings. Reviews, opinion pieces, and other publication types that do not report original empirical findings will be excluded.

### Search Strategy

Searches will be conducted in Medline, EMBASE, and PsycINFO databases. An example search string developed for Ovid PsycINFO is shown in Table 1, which will be replicated for EMBASE and Medline databases. Reference lists of selected articles will also be hand searched to identify additional relevant articles not captured by the initial search strategy.

**Table 1:** Sample search strategy developed for Ovid PsycINFO.

Search	Terms
1	SH^a^: Latent Variables/ or Latent Class Analysis/ or Latent Profile Analysis/ or Item Response Theory/ or Principal Component Analysis/
2	(general factor* or p-factor* or transdiagnostic* or psychopathology network* or symptom network* or bridge symptom* or comorbidity network* or latent* or factor mixture model* or multimode* or item response theory).mp^b^.
3	1 or 2
4	SH: exp^c^ Psychopathology/ or exp Psychiatry/ or exp Dual Diagnosis/ or exp Comorbidity/
5	(psychopatholog* or psychiatr* or comorbid* or co?occur* or dual diagnos*).mp.
6	4 or 5
7	3 AND 6
8	(Child* or adolescen* or teen* or youth* or pediatr* or paediatr* or young or emerging adult* or youth).mp.
9	SH: exp risk factors/ or exp protective factors/
10	((risk or protec* or resilienc* OR underlying or vulnerab*) adj4 (factor* or mechanism* or character*)).mp.
11	9 OR 10
12	7 AND 8 AND 11
13	Limit 12 to English language
14	Limit 13 to peer-reviewed journals

^a^SH: subject heading.

^b^mp: title, abstract, original title, name of substance word, subject heading word, keyword heading word, protocol supplementary concept word, rare disease supplementary concept word, unique identifier, synonyms.

^c^exp: explode.

### Selection of Studies

All titles and abstracts will be screened by one reviewer (SJL); the other reviewers (CC, NN, MS) will screen 25% of the titles and abstracts, which will be randomly selected. For all studies identified in the initial screen, the full-text articles will be reviewed and assessed against the eligibility criteria by two reviewers (SJL and MS). Disagreements at each stage of screening will be resolved through discussion or by a third reviewer (CC). A PRISMA flow chart will be created to show the results of each stage of the screening process.

### Review Procedure and Data Extraction

Citations will be imported into the Covidence systematic review software [24], which will be used to remove duplicates and screen tittles, abstracts, and full texts. The following information will be extracted by the primary reviewer (SJL): publication details (authors, year of publication, country), study design (eg, cross-sectional, longitudinal), sample characteristics (sample size, mean age, ethnicity, sex), psychopathology measures (measures used), informant type (parent, self, other), risk or protective factor measures, data analysis strategy (techniques used, model specification, indicator type), outcome statistics (eg, test statistics, *P* values, effect size, model fit statistics, network centrality statistics). For longitudinal studies, additional information will be extracted regarding follow-up intervals and frequency. A summary of main findings will also be recorded.

### Assessment of Quality

Following data extraction, study quality will be assessed independently by two reviewers. Cross-sectional studies will be evaluated using the Joanna Briggs Institute Critical Appraisal Checklist for Analytical Cross-Sectional Studies, and longitudinal studies will be evaluated using the Joanna Briggs Institute Critical Appraisal Checklist for Cohort Studies [25].

## Results

This systematic review is ongoing. At the time of submission, full-text screening was completed, and hand searching of articles for additional studies to be included was underway. Findings will be summarized in a narrative synthesis and grouped by research domain, such as genetic, neurobiological, cognitive, social, environmental, or any other broad themes that emerge from the review. Studies will also be summarized by statistical approach. Analysis of subgroups or subsets will be determined based on results of the review and availability of sufficient data. Results are expected to be completed by the end of 2020.

## Discussion

Understanding how risk and protective factors are associated with empirical models of psychopathology is critically important to determining which factors will be most useful to target when developing treatment and preventative interventions. It may be that some transdiagnostic risk factors are associated with a general vulnerability to all mental disorders, while others may be more specific to certain dimensions or spectra (eg, internalizing, externalizing). Factors associated with a general liability may serve as fruitful targets for preventative interventions, whereas specific factors may be more useful in developing selective or indicated interventions.

The results of this systematic review will provide a much-needed critical analysis of the risk and protective factors for mental and substance use disorders in young people derived from empirically based models of psychopathology. Findings will help guide and accelerate the development of transdiagnostic prevention programs. To our knowledge, this will be the first systematic review of the risk and protective factors associated with empirically based models of psychopathology in young people. The critical synthesis of this evidence provides an opportunity to better understand the factors that contribute to the onset and maintenance of psychopathology in young people. This information can provide a foundation upon which interventions can be designed that are better able to prevent or reduce mental health problems and in turn disrupt the cascade of psychopathological sequelae into adulthood.

## Appendix

Multimedia Appendix 1 PRSIMA-P Checklist 2015.

## Abbreviations

HiTOP: Hierarchical Taxonomy of Psychopathology
PECO: Population Exposure Comparator Outcome
PRISMA-P: Preferred Reporting Items for Systematic Reviews and Meta-Analysis Protocols

## References

[1] Whiteford HA, Ferrari AJ, Degenhardt L, Feigin V, Vos T (2015). The global burden of mental, neurological and substance use disorders: an analysis from the Global Burden of Disease Study 2010. PLoS One.

[2] Costello EJ, Copeland W, Angold A (2011). Trends in psychopathology across the adolescent years: what changes when children become adolescents, and when adolescents become adults?. J Child Psychol Psychiatry.

[3] Kessler RC, Ormel J, Petukhova M, McLaughlin KA, Green JG, Russo LJ, Stein DJ, Zaslavsky AM, Aguilar-Gaxiola S, Alonso J, Andrade L, Benjet C, de Girolamo G, de Graaf R, Demyttenaere K, Fayyad J, Haro JM, Hu CY, Karam A, Lee S, Lepine J, Matchsinger H, Mihaescu-Pintia C, Posada-Villa J, Sagar R, Ustün TB (2011). Development of lifetime comorbidity in the World Health Organization world mental health surveys. Arch Gen Psychiatry.

[4] Shanahan L, Copeland W, Costello E Jane, Angold A (2008). Specificity of putative psychosocial risk factors for psychiatric disorders in children and adolescents. J Child Psychol Psychiatry.

[5] Leadbeater B, Thompson K, Gruppuso V (2012). Co-occurring trajectories of symptoms of anxiety, depression, and oppositional defiance from adolescence to young adulthood. J Clin Child Adolesc Psychol.

[6] Kotov R, Krueger RF, Watson D, Achenbach TM, Althoff RR, Bagby RM, Brown TA, Carpenter WT, Caspi A, Clark LA, Eaton NR, Forbes MK, Forbush KT, Goldberg D, Hasin D, Hyman SE, Ivanova MY, Lynam DR, Markon K, Miller JD, Moffitt TE, Morey LC, Mullins-Sweatt SN, Ormel J, Patrick CJ, Regier DA, Rescorla L, Ruggero CJ, Samuel DB, Sellbom M, Simms LJ, Skodol AE, Slade T, South SC, Tackett JL, Waldman ID, Waszczuk MA, Widiger TA, Wright AGC, Zimmerman M (2017). The Hierarchical Taxonomy of Psychopathology (HiTOP): A dimensional alternative to traditional nosologies. J Abnorm Psychol.

[7] Dalgleish T, Black M, Johnston D, Bevan A (2020). Transdiagnostic approaches to mental health problems: Current status and future directions. J Consult Clin Psychol.

[8] Teesson M, Baker A, Deady M, Mills K, Kay-Lambkin F, Haber P, Baillie A, Christensen H, Birchwood M, Spring B, Brady K, Manns L, Gournay K (2014). Mental health and substance use: opportunities for innovative prevention and treatment: Report prepared for the Mental Health Commission of NSW.

[9] Forbes MK, Rapee RM, Krueger RF (2019). Opportunities for the prevention of mental disorders by reducing general psychopathology in early childhood. Behav Res Ther.

[10] Harvey A, Watkins E, Mansell W, Shafran R (2004). Conclusions. Cognitive behavioural processes across psychological disorders: A transdiagnostic approach to research and treatment.

[11] Eaton NR (2015). Latent variable and network models of comorbidity: toward an empirically derived nosology. Soc Psychiatry Psychiatr Epidemiol.

[12] Krueger RF, Markon KE (2011). A dimensional-spectrum model of psychopathology: progress and opportunities. Arch Gen Psychiatry.

[13] Fried EI, van Borkulo CD, Cramer AOJ, Boschloo L, Schoevers RA, Borsboom D (2016). Mental disorders as networks of problems: a review of recent insights. Soc Psychiatry Psychiatr Epidemiol.

[14] Achenbach TM (1966). The classification of children's psychiatric symptoms: a factor-analytic study. Psychol Monogr.

[15] Caspi A, Houts RM, Belsky DW, Goldman-Mellor SJ, Harrington H, Israel S, Meier MH, Ramrakha S, Shalev I, Poulton R, Moffitt TE (2014). The p Factor: One General Psychopathology Factor in the Structure of Psychiatric Disorders?. Clin Psychol Sci.

[16] Borsboom D (2017). A network theory of mental disorders. World Psychiatry.

[17] Crews SD, Bender H, Cook CR, Gresham FM, Kern L, Vanderwood M (2017). Risk and Protective Factors of Emotional and/or Behavioral Disorders in Children and Adolescents: A Mega-Analytic Synthesis. Behavioral Disorders.

[18] McMahon SD, Grant KE, Compas BE, Thurm AE, Ey S (2003). Stress and psychopathology in children and adolescents: is there evidence of specificity?. J Child Psychol Psychiatry.

[19] Forbes MK, Kotov R, Ruggero CJ, Watson D, Zimmerman M, Krueger RF (2017). Delineating the joint hierarchical structure of clinical and personality disorders in an outpatient psychiatric sample. Compr Psychiatry.

[20] Moher D, Shamseer L, Clarke M, Ghersi D, Liberati A, Petticrew M, Shekelle P, Stewart LA, PRISMA-P Group (2015). Preferred reporting items for systematic review and meta-analysis protocols (PRISMA-P) 2015 statement. Syst Rev.

[21] Lynch SJ, Sunderland M, Newton NC, Chapman C (2020). Systematic Review: Transdiagnostic Risk and Protective Factors for Psychopathology in Young People. Center for Open Science.

[22] Morgan RL, Whaley P, Thayer KA, Schünemann HJ (2018). Identifying the PECO: A framework for formulating good questions to explore the association of environmental and other exposures with health outcomes. Environ Int.

[23] (2014). Recognizing Adolescence. World Health Organization.

[24] (2020). Covidence systematic review software.

[25] Moola S, Munn Z, Tufanaru C, Aromataris E, Sears K, Sfetic R, Currie M, Lisy K, Qureshi R, Mattis P, Mu P, Aromataris E, Munn Z (2017). Chapter 7: Systematic reviews of etiology and risk. JBI Reviewers Manual.

